# Supplementary material for: Human flourishing and religious liberty: Evidence from over 150 countries
Source: PLoS One. 2020 Oct 1;15(10):e0239983. doi: 10.1371/journal.pone.0239983 (PMC7529430; doi:10.1371/journal.pone.0239983)
Supplement: Attachment — Submitted filename: Response to Reviewers.pdf [file pone.0239983.s001.pdf]

Dear Yanyan Gao and anonymous referees,

Thank you so much for taking the time to review this article and offer many good suggestions. Among many other adjustments, the two main areas of focus in the this revision include: increased clarity about the contribution, relative to existing literature on the cross-section between religious liberty and economic development, and improved discussion (and implementation) of the causal strategy, which includes additional controls to address concerns about omitted variables.

Below, I offer a point-by-point reply to each of the ideas that the referees raised.

Reviewer #1

Grim, Clark and Snyder's 2014 study actually went beyond correlational relationship between religious freedom and economic growth, empirically testing and finding the tandem effects of religious restrictions and hostilities to be detrimental to economic growth while controlling for other theoretical, economic, political, social, and demographic factors. This should be specifically mentioned, especially since your findings are in line with those and expand them beyond mere GDP growth to human flourishing. (see attached file)

> Reply: Thank you for drawing my attention to these controls, which go a long way to help deal with possible omitted variables. To address this concern, I introduced the sentence: "While these studies have generally controlled for various country-specific factors, ranging from tax rates to economic activity, such a correlation could still reflect confounding factors that prevent a causal interpretation." I have also adjusted some of the other language throughout the draft to point out examples (unemployment, FDI, property rights).

In your final section, it would be very helpful to split out the "conclusions" from "understanding the mechanisms" by having as the conclusion a bulleted summary of your main findings and their implications for societies.

> Reply: Great point. I separated out a final section for conclusions and policy implications. I did not put bullet points for stylistic considerations, but now the implications are clear.

You mention the importance of China being a respected democratic partner as your very last point. However, since your whole paper is about flourishing, I strongly suggest your comment on China should focus on pointing out that if the Chinese Communist Party (i.e., not 'China' – the two are not synonymous) were to make religious freedom a priority and a reality for all, it would be a significant asset to China's economic recovery and the flourishing of its people following the pandemic. Moreover, such a move would also help address the growing global backlash toward China following the pandemic.

> Reply: Thank you—important distinction. The tie into the current situation is a great idea! Specifically, here's the last sentence: "Moreover, given the unfolding COVID-19 pandemic and the international backlash against their failure to warn against the spread of the virus, the CCP has a unique opportunity to not only advance its own economic development through an expansion of religious liberty, but also signal to the international community that it is willing to make reasonable concessions on important human rights issues."

Regarding the backlash, see: "Coronavirus: how will China's role in the global economy change when faced with pandemic backlash?" Zhou Xin, 28 Apr, 2020, SCMP, https://www.scmp.com/economy/china-economy/article/3081778/coronavirus-how-will-chinas-role-global-economy-change-when

Reply: Good read—digested that in forming the new conclusion.

Reviewer #2

I suggest four major themes for improvement. First, the causal claims that this paper makes need to be argued better. The introduction on page 4 introduces the reader to the three possible mechanisms by which religious liberty could impact well-being. However, their expansion in Section 5 'Understanding the mechanism and conclusion' (pp.20-21) is not convincing. The first example of the link between religious factors and the emergence of democracy, does not show how religious plurality or religious freedom contributes to democracy. Instead it presents evidence of a specific religious movement contributing towards democracy. The second example connecting religious plurality and educational attainment is relatively more convincing. In case of the third causal mechanism, again the examples cited reflect the fact that specific religious movements contributed to the promotion of peace and positively influenced civil society. There is a body of literature that points to the link between religion and pro-social behavior, however, the focus of this study is not religiosity rather it is religious pluralism. Thus, there is a strong need for the causal claims to be supported and positioned better.

Reply: Thank you–obtaining a causal estimate is indeed the most challenging component of the paper. I have revised the text away from the role of religious affiliation and towards religious plurality. The argument is that plurality allows for a multiplicity of ideas and mutual respect, which are integral for a functioning democracy and civic discourse. The section has been rewritten to distinguish religious affiliation and liberty. I have also reworked the discussion of causal inference throughout the draft. My impression is that the battery of evidence paints a clear picture, ranging from the FE/IV to heterogeneity analyses.

As will become clear in an upcoming comment, the IV estimate is also much closer to the baseline result, which gives greater confidence of causality. I am especially thankful for your pushing on identification since the key to obtaining the new estimate was the inclusion of additional cross-country controls that are correlated with the deployment of missionaries.

Second, the level of detail the paper provides with respect to the data and methodology and the organisation of this information needs to be improved upon considerably. This begins from the abstract itself, where it is not clear how many countries feature in the first part of the analysis and which is the time-period under consideration. Is it the same set of countries that are used in the analysis that are then used in the second part of the analysis that uses micro-data?

Reply: Fair points—these important details are now introduced. The World Poll covers 154 countries, but not every country is surveyed every year—only 47% are surveyed each year. However, since the baseline specification exploits within-country variation, that's not an identification concern, particularly since 80.6% are surveyed at least 11 times. Nonetheless, restricting the sample to a panel countries observed at least 11 times produces a marginal

effect of 0.025 (p- value = 0.014), in comparison with 0.024 (p-value = 0.004). Moreover, the coefficient is 0.028 (p-value = 0.012) with countries observed at least 12 times and 0.022 (p-value = 0.064) with countries observed all 13 years. I also found that the correlation between the number of times a country is observed and the share of households that are thriving is 0.10, suggesting that any measurement error is probably not correlated with unobserved heterogeneity in the outcome variable. These details are added.

I have omitted the number of times a country is observed from the abstract (to conserve on space). However, I have also flagged the caveat in the introduction, as well as the measurement section and an additional footnote in the results section.

The discussion of the mechanisms uses a broader sample of countries, but the results are economically invariant to restricting the sample to those observed in the World Poll. The only difference is that the estimates become less precise when more countries are dropped, which is in part a function of the fact that there is not a significant amount of year-to-year variation in these institutional variables. However, when country and year fixed effects are not included (exploiting more of the cross-section), the results are indistinguishable with the sample restrictions. For example, a 1sd rise in religious liberty is associated with a 0.30sd decline in public corruption when the sample is restricted to countries that were observed at least 11 times in the World Poll sample (and the baseline effect is 0.31). As long as the two sources of data are representative, then slight difference in the included countries will not make a big difference, especially given the inclusion of country fixed effects.

The introduction gives the reader some semblance of the time-period of analysis (2006-2018). But it is still unclear how many countries are part of this analysis and which dataset is used in the first part of the analysis and which in the second. Providing this information upfront in the introduction itself is important, as the paper states (on page 6) that one of its contributions is that unlike past studies which use the World Values Data, this particular study employs a much more comprehensive dataset.

Reply: Thank you—good point and my correction above also addresses it here.

On page 11, Table 3 mentions "agricultural share" and "services share", but it is unclear whether this implies the sectoral share with respect to the GDP or with respect to the labor force. It is only much later on page 17, where it is specified that agricultural share means agricultural employment share.

Reply: Sorry for the imprecision. While the text and table notes describes it as "employment shares," I omitted "employment" from the table so that the label is not too long (purely for aesthetic reasons). Moreover, I used employment shares since measurement of GDP in African countries is less imprecise; see, for example, Young (2012).[1]

On page 14, the paper states: "Moreover, to address the possibility that political reforms coincide with economic reforms, proxies for economic freedom from the Heritage Foundation's Index of Economic Freedom are included as controls." It would be good to mention what these proxies are.

---

[1] Young, Alwyn. 2012. :"The African growth miracle." Journal of Political Economy, 120(4): pp. 696-739.

Reply: Fixed—these include an index of property rights, business freedom, and labor freedom. Moreover, I have added additional explanation of these indices into the data and measurement section so that readers understand how they are constructed.

Another example, on page 15, the study states: "Drawing on his measure of missionary exposure, measured through both Protestant and Catholic missionaries per 10,000 individuals in 1923, Figure 6 plots their relationship with religious liberty averaged between 2006 and 2018, producing correlations of 0.27 and 0.33, respectively." This statement does not shed light on essential information such as the source of this data, nor does it tell the reader the geographic coverage or time-period of reporting. I had to read through the footnote of Table 6 (on page 41) to find the answer to my question. It would be more effective if this information were to be reported in the main text itself.

Reply: Sorry for the miscommunication. I was relegating much of the data description to the citation to his seminal paper, but it is good to include here too for readers. I added more of it and also include a citation to a forthcoming paper by Woodberry that goes in more detail.

Finally, Table 5 is supposed to report the OLS and fixed effects estimates. It is not clear which column refers to which. A little more attention to detail would have been good.

Reply: Sorry for the confusion—you are right, I accidentally forgot to label the even numbered columns that have country and year FE. Fixed!

Third, this point relates to the policy implications that the study draws. According to the paper the causal mechanism by which increases in religious liberty lead to increases in well-being is through the links between religious liberty (social capital) and democracy. But then this very causal claim would belie the policy implication that the study cites by giving the example of China. If religious liberty is related to increases in democratic governance, would authoritarian countries like China be interested in pursuing it? Moreover, what would explain the case of a country like Singapore where religious liberty co-exists with a lack of an actual democracy? But while the latter question may be a topic for future research, the current study might need to give some thought to the policy implications it draws. Perhaps it would be more convincing to draw policy implications around the premise that if religious liberty helps increase human flourishing, then the recent decline of religious liberty in countries that rank higher in property rights and the rule of law, does not bode well.

Reply: Exactly, and that's precisely what I think the takeaway should be too. In particular, having a thriving society is impossible when people feel like their ideas are suppressed. I have added additional discussion about how declines in religious liberty generally precede declines in economic freedom and economic activity. That is intuitive: the minute the government gets more restrictive with how people can believe and think, then people are also less willing to take risk as entrepreneurs, property owners, etc, since they realize they cannot trust the government and are unwilling to put themselves out there.

Fourth, this point is with respect to the analysis. I have some questions related to the controls, the instrumental variable and the spatial-temporal coverage of the analysis. Some minimum rationale for the controls needs to be provided. For instance, what is the justification for including specifically the share of agriculture and services as controls, in the main regressions. Why not industry and services?

Reply: Great reminder. I have added additional discussion and rationale for the controls. The main constraint is data. While some countries have measurement of industry composition, the more detailed the controls, the more countries drop out during inference, particularly in developing countries where there is some of the greatest variation in religious liberty.

Some recent papers have also questioned the supposed exogeneity of missionary activity and its use an instrumental variable. Jedwab et al. (2018) argue that missionary's locational activity decisions were driven by economic factors as missionaries went to healthier, safer and more accessible and developed areas first. Thus, locations with past missions tended to be more developed and this bias is further enhanced as historical mission atlases tend to only report the best mission locations. The same study also quotes a missionary Thauren in Togo who describes the settlement strategy as one where every effort is made to acquire knowledge of the region (p.2). Evidence such as this lowers the plausibility of the instrument, but if perhaps the article were to support its instrumental variable strategy with some basic tests for IVs such as by quoting the Stock-Watson statistics etc., it would make the results more believable.

Reply: Thank you for flagging this paper. I was not aware of the paper and the citation and a lengthy discussion is included in the text. Jedwab et al. (2018) make an important point about measurement—many missions might not have been included in some of the atlases used to construct the data used as an instrument. They have also done great work assembling such comprehensive data, although it is just restricted African countries (mainly Ghana).

I summarize my edits in the revision before discussing their paper. I omit the IV table and include a discussion in the text to under-emphasize the IV results for the concerns that you flagged. Moreover, I control for geographic characteristics (whether the country is an island, whether it is landlocked, and latitude), average temperature in the hottest and coldest month, and whether there was a malaria epidemic in the country, on top of the standard individual and contemporaneous country controls that are in the baseline specification. These controls address the possibility that certain countries might have had better transportation networks or a lower incidence of disease, which could affect selection into them (Jedwab et al., 2018). The F-statistic is only 7, which is slightly below the rule-of-thumb equal to 10, but is close enough that I interpret the results as complementary to the baseline specification.

Now, let me highlight three major concerns associated with Jedwab et al. (2018), which are also brought up in new work by Woodberry (2020), that validate the IV approach.[2]

First, the 90% missing mission stations Jedwab et al. (2018) report are almost entirely local churches staffed by Ghanaians, meaning that they do not have foreign missionaries at them. Before local denominations got their independence, a mission (an Akan going to witness to Ashanti) and a church (an Akan setting up a cocoa plantation in a neighboring area and setting up a church for his family and workers) is "fuzzy"—both would be part of the mission umbrella organization. The focus of Woodberry (2012), however, is on foreign

[2] Woodberry, Robert D., "The Future of the Past: Measuring the Persistent Impact of Protestantism on Democracy and Its Diffusion to Other Religious Traditions", British Journal of Political Science forthcoming (2020).

missionaries, not mission stations, or mission stations/local congregations. Jedwab et al. study a separate, but nonetheless important, question—distinct from foreign missionaries.

Second, their data sources have some challenges and omissions. They evaluate *A Geography and Atlas of Protestant Missions* (Beach 1903), which was compiled primarily by a single scholar, and the *Ethnographic Survey of Africa* (Roome 1925), which was not compiled by a missionary organization and contains no Catholic mission stations in Southern Africa. Surprisingly, they do not evaluate any of the atlases compiled by the International Missionary Council with other international missionary conferences. Woodberry used some of these (e.g., the World Missionary Atlas for the Protestant data or the Atlas Hierarchicus for the Catholic Data) since they are higher quality than earlier atlases. (For example, the World Missionary Atlas was funded by Rockefeller and has been described as "near to perfect").

Third, Jedwab et al. overlook several fundamental institutional features. For example, the Basel Mission began introducing cash crops into the local economy and training Ghanaians how to grow them in 1843. They and other missionaries wanted to introduce "legitimate" trade to undermine the slave trade and the local practice of slavery, as well as to produce a self-sustaining church. To introduce cash crops and export crops to the international market (through a trading company they created), the Basel Mission introduced the first cocoa plants to Ghana in by at least 1857. Few Africans initially planted cocoa and this early effort was put on hold by an Ashanti invasion of the area. The Ghanaian often credited with popularizing cocoa planting was Tetteh Quarshie (who brought seeds from Fernando Po where he worked on a plantation for 6 years), but he was trained as an apprentice at the Basel Mission where cocoa was initially introduced and was a committed Christian. The vast majority of those who spread cocoa farming across Ghana were Christian Akwapem farmers and during this second phase of cocoa introduction the Basel Mission actively imported seeds and trained Africans in how to grow, harvest, and process cocoa.

In this sense, historical evidence suggests that missionaries and their converts spread cash crops, imported workers and planted churches because they were committed Christians and wanted a place for them and their workers to worship—economic development was not random. (This is also visible in many of their statistical tables with missions being associated with later or concurrent growth, although Jedwab et al. assume this is because other types of growth caused the missions rather than the other way around.) Similar historical patterns in Nigeria and other countries with missionaries also abound.

These considerations are not to negate the work from Jedwab et al. (2018), but to point out that this is an ongoing debate and there are many valid counterarguments to their results and replies to the concerns that they raise about Woodberry (2012) and others.

Finally, how many of the countries from the original analysis feature in the instrumental variable analysis? Also, the first two parts of the analysis use a dataset between 2006-2018, but in tracing the causal mechanisms the time-period of analysis is from 1990-2018. Is there a particular reason why a different time-period was chosen for this part of the analysis?

Reply: The limitation comes from the World Poll, which only began in 2006. If the sample for the mechanisms section is restricted to 2006-2018, the same results flow through, but the

standard errors are larger. See, for example, Table XX in the Online Appendix. I have now footnoted the difference in sample selection as a data constraint.

1. Page 6: "98% of the world's population", the author probably means 150 countries that make up 98% of the world's population. But worded the way it is at present, it seems as if the World Gallup Poll surveys 98% of the world population.

Reply: Great catch!

2. Pages 6-7: What is the average sample size that Gallup uses for a country? It would be useful to present this information.

Reply: It really depends on the country. For example, the median number of respondents for a country-year pair is 949. However, the mean is even higher: 1,240 due to China and India that have 6,145 and 11,420 respondents in certain years (specifically 2012). However, the minimum sample size is 290 and the 5th percentile is 776 respondents.

3. It is mentioned that the V-Dem dataset measures responses on a 0-4 scale and then rescales them. It would be good to mention the new scale in the text.

Reply: The index ranges between -3.639 and 2.798 based on the assumptions about normality, but these values do not really mean much—that's why it is important to create the standard normal version when considering the regression (mean zero, sd of one).

4. Page 8: "In this sense, while the Gallup data provides several comparable measures to the WVS, it also provides a wide range of additional responses about institutions, human flourishing, and religious affiliation." In what sense does it provide information on institutions?

Reply: They ask about institutional trust and confidence, e.g., the government. However, since I am not using these additional data points, I did not want to distract readers.

5. Page 8-9: Pew research centre data is mentioned here. Its mention here necessitates an explanation for what it does that makes it inferior to the V-dem dataset.

Reply: Fixed! The comparison was done for benchmarking—unfortunately the Pew data is only cross-sectional, meaning that there is no longitudinal variation to exploit.

6. There is some repetition on pages 9 and 10.

Page 9: "Moreover, because experts provide ratings across more than one categories of questions, those who systematically deviate from the norm will receive a lower reliability rating, which leads to a lower weight in the construction of the overall index."

Page 10: "Because experts in the V-Dem survey answer multiple questions, multiple

answers that deviate from the norm will produce a lower reliability, thereby generating lower weights in the inclusion of the respondent's answer in the overall score."

Reply: Thanks for flagging—originally was used just for emphasis since this is a unique approach to measuring institutions across countries, but you're right: it looked odd! Fixed.

7. Page 11: How does pew research centre define restrictions and social hostilities?

> Reply: The Pew data is fairly well-known, but I have included some additional explanation. The citation is included so that interested readers can go to the source, rather than take up extra space in the text. As an example, social hostilities is included below.
>
> *The Social Hostilities Index is based on 13 questions (see the Summary of Results) used by the Pew Forum to gauge hostilities both between and within religious groups, including mob or sectarian violence, crimes motivated by religious bias, physical conflict over conversions, harassment over attire for religious reasons, and other religion-related intimidation and violence, including terrorism and war (see the Methodology). Several of these questions allow for gradations of severity. In addition, there is some overlap among questions that measure mass violence – for example, killings picked up by Question No. 2, "Was there mob violence related to religion?" might also be picked up by Question No. 5, "Was there a religion-related war or armed conflict in the country?" – which serves to give more weight in the index to the most extreme consequences of religious hostilities, such as deaths and the displacement of people from their homes.*

8. Page 11: "There are many approaches for measuring economic freedom, most notably the Index of Economic Freedom [46]". Does this refer to the Heritage Foundation Index of Economic Freedom?

> Reply: Correct—I fixed the language. I also added two citations to help make the case for these different indices. Fraser is perhaps "more common," but my preference is over the Heritage Index. The advantage of Fraser is that it is a longer time series, but that is a moot point in my situation since only 2006 and after matter.

9. Page 17: "Moreover, the agricultural employment share is negatively correlated with human flourishing, which reflects the fact that lower income countries have greater agricultural employment shares [57]". This sentence could be worded to make it more clear. Is the point that countries with higher agricultural employment shares are those where human flourishing is reported to be lower, these also tend to be low income countries?

> Reply: Good catch—yes, it's just that these countries are at an earlier stage of economic development. I made the clarification in the revision.

10. Pages 17 and 23, use the term probability in interpreting the results. For example on page 17: ""Columns 4 and 12 present the baseline specification that contains the standard controls and fixed effects on both country and year, suggesting that 1sd rise in religious liberty is associated with a 0.03pp rise in the probability that an individual is thriving and a 0.08sd rise in individual well-being". Is that correct?

> Reply: Correct. When the outcome variable is the 1/0 indicator for whether the person is thriving, then the marginal effect is interpreted as a probability. However, when the outcome variable is the z-score of well-being, then it is interpreted as a standard deviation.

11. Page 19: "This suggests that, while religious individuals benefit overall from improvements in religious liberty, the benefits are concentrated among Christians." This is confusing because the preceding and subsequent sentence mention Christians or Jews together. And then this sentence restricts its scope to Christians.

Reply: Thanks! I meant to include "and Jews" at the end—fixed. The only limitation here is that there are some countries that are also fairly restrictive against Muslims, like the CCP in China. However, respondents in China generally do not report their religion in China since the government is so controlling over any survey efforts, so China is not even included in that regression since these respondents do not report their religion.

12. Pages 34-35: Tables 1 and 2 have a problem with the labeling.

Reply: Think it's looking better now that it is in PLOS One format, but feel free to flag if something still looks off.

13. Page 34: Table 1's footnote seems incomplete. It does not cover the case of questions where there are 11 items, for example the "Daily Experience Index".

Reply: Great catch—had dropped off the positive/negative experience terms.

14. Page 35: It would be helpful if for Table 2, the table/ appended note mentioned the precise question that was asked.

Reply: Good call. I added it—very short question text: "is there freedom of religion?."

15. Pages 33-42: There are issues with the order of the tables and figures. If they are supposed to appear in the order they are mentioned in the text, then table 3 is out of place.

Reply: This is getting corrected in the PLOS One formatting. The latex structure was set to fill in tables/figures wherever they could fit after a certain point, so they were not always positioned in the exact spot. Hopefully this fixes it!

16. Where does the data for the estimations in Section 5 come from i.e. the data on armed conflict etc.? Is it from V-dem?

Reply: Correct, it also comes from V-Dem.
